# Diffuse Cutaneous Systemic Sclerosis With Normotensive Scleroderma Renal Crisis and Myopericarditis: A Case Report

**DOI:** 10.7759/cureus.78858

**Published:** 2025-02-11

**Authors:** Luís Augusto Barbosa Franco Zörrer, Lucas Yugi de Souza Terui, Rodrigo Fanini Balena, Ana Luisa Woidello Miyazima, Rafael Miyazima

**Affiliations:** 1 Internal Medicine, Clinical Hospital Complex of the Federal University of Paraná, Curitiba, BRA

**Keywords:** myopericarditis, scleroderma, scleroderma renal crisis, small vessel vasculopathy, systemic sclerosis

## Abstract

Systemic sclerosis (SSc) is a rare autoimmune disease characterized by fibrosis and multi-organ dysfunction, primarily affecting the heart, lungs, and kidneys. Scleroderma renal crisis (SRC) can present as hypertensive or normotensive, with the latter being more challenging to diagnose due to the absence of hypertension at onset. Normotensive SRC carries a worse prognosis, with an increased risk of renal failure and a poor response to treatment. The presence of cardiac complications, such as myopericarditis, further exacerbates the clinical course, creating significant management challenges. Moreover, hypotension in normotensive SRC complicates therapeutic interventions, particularly the use of angiotensin-converting enzyme (ACE) inhibitors.

This case report highlights a patient with diffuse cutaneous systemic sclerosis (dcSSc) who presented with normotensive SRC complicated by myopericarditis, resulting in acute renal and heart failure. It underscores the need for early recognition of this rare form of renal crisis, especially when accompanied by cardiac complications, given its atypical presentation without significant hypertension at onset. The report emphasizes the critical importance of identifying risk factors and addressing the challenges of managing normotensive SRC to improve patient outcomes.

## Introduction

Systemic sclerosis (SSc) is a rare autoimmune disease marked by small vessel vasculopathy, excessive autoantibody production, and fibroblast dysfunction [1]. Its prevalence ranges from 38 to 341 per million, with a higher incidence in women [2]. SSc presents with variable clinical manifestations, commonly including cutaneous thickening and involvement of internal organs such as the heart, lungs, and kidneys [2,3].

SSc can be classified into clinical subsets: limited cutaneous systemic sclerosis (lcSSc), typically associated with anti-centromere antibodies and skin thickening distal to the elbows and knees; diffuse cutaneous systemic sclerosis (dcSSc), linked to anti-topoisomerase I and anti-RNA polymerase III antibodies, presenting more severe internal organ involvement and shorter survival; and sine scleroderma, without cutaneous involvement [1-3].

One of the most severe complications of SSc is scleroderma renal crisis (SRC), characterized by acute kidney injury and abrupt onset of hypertension. The exact pathophysiology remains unclear but likely involves intimal thickening of renal interlobular and arcuate arteries, leading to vascular narrowing and reduced renal perfusion, which stimulates juxtaglomerular apparatus hyperplasia and increased renin release. SRC occurs in 1-2% of patients with lcSSc and 5-20% of those with dcSSc [4-6].

Risk factors for SRC include diffuse skin involvement, rapid progression of skin thickening, presence of anti-RNA polymerase III antibodies, high-dose corticosteroid use, pericardial effusion, and disease duration of less than four years from SSc onset [3,6-11]. Heart failure is observed in 40% of SRC cases.

A variant called normotensive SRC, occurring in about 10% of cases, is linked to a worse prognosis and an increased risk of requiring chronic dialysis, likely due to delayed diagnosis [9-11]. We present a case of normotensive SRC with concurrent myopericardial involvement in a patient with newly diagnosed dcSSc.

## Case presentation

A 47-year-old female patient, diagnosed with dcSSc 18 months prior to admission, experienced rapidly progressive skin thickening and systemic involvement, including interstitial lung disease (ILD), esophageal involvement, and frequent Raynaud's phenomenon. The initial clinical symptoms included generalized pruritus, lesions on the ears and elbows, hand edema, and carpal tunnel syndrome. In the initial management at an external hospital, the patient was treated with methotrexate from February to August 2023, with gradual titration up to 15 mg per week, but without a satisfactory response. She also received prednisone (20 mg/day) for four months, primarily for the management of carpal tunnel syndrome. Upon admission, she was on mycophenolate mofetil 500 mg three times daily.

Previous laboratory tests showed a positive antinuclear antibody (ANA) with a mixed pattern (anti-DNA and anti-topoisomerase I), exhibiting high titers (>1:640) and elevated anti-topoisomerase I (1:240). A chest computed tomography (CT) scan performed eight months before admission revealed early ILD, characterized by discrete subpleural reticular infiltrates, indicating mild pulmonary involvement. 

A baseline electrocardiogram (ECG) performed eight months prior to admission showed no abnormalities. Transthoracic echocardiography (TTE) performed seven months prior to admission showed preserved systolic function, with a left ventricular ejection fraction (LVEF) of 67%, and no evidence of pulmonary hypertension.

Over the past six months, the patient’s symptoms progressed, including worsening dyspnea, fatigue, and limitations in daily activities. She also reported transfer dysphagia over the last three months, requiring dietary adjustments to processed foods. Two days prior to admission, the patient experienced worsening dyspnea and pleuritic chest pain. She was evaluated at an external hospital, where she received 500 mg of intravenous (IV) hydrocortisone for symptomatic management.

On September 17, 2023, the patient was admitted to the emergency department due to a significant worsening of dyspnea over the previous two days, classified as grade 3 on the Modified Medical Research Council (mMRC) scale. This was accompanied by a dry cough and ventilator-dependent retrosternal chest pain. 

On physical examination, the patient was alert, oriented, well-perfused, and hydrated, with an oxygen saturation of 94% on room air. She was normotensive (125/85 mmHg) and in generally good condition, though dyspneic. Pulmonary auscultation revealed diffuse velcro crackles in all lung fields. A cardiac examination showed normal heart sounds, without murmurs. The abdomen was peristaltic, tympanic, and non-tender on palpation, and no edema was observed in the lower extremities.

The dermatological examination revealed diffuse skin thickening, with a Rodnan score of 20, sclerodactyly, and 'salt-and-pepper' hyperpigmentation on the hands and back. Telangiectasias were observed on the digital pads and malar region, without signs of digital ulcers or stellate scars. Additionally, the patient presented with microstomia and xerostomia.

The initial laboratory tests revealed leukocytosis (16.1 K/µL) and a notable increase in creatinine, rising from a baseline of 0.75 mg/dL to 1.51 mg/dL upon admission, indicative of stage 2 acute kidney injury according to the Kidney Disease: Improving Global Outcomes (KDIGO) classification. Additionally, elevated blood urea nitrogen (BUN) levels (40 mg/dL) and high-sensitivity C-reactive protein (hs-CRP) levels of 3.91 mg/dL were observed (Table 1).

**Table 1:** Laboratory tests at admission. MCV: mean corpuscular volume; MCH: mean corpuscular hemoglobin; MCHC: mean corpuscular hemoglobin concentration; RDW: red cell distribution width; WBC: white blood cells; BUN: blood urea nitrogen; hs-CRP: high-sensitivity C-reactive protein; pCO2: partial pressure of carbon dioxide; HCO_3_: bicarbonate; INR: international normalized ratio; DB: direct bilirubin; IB: indirect bilirubin; BT: total bilirubin; ALP: alkaline phosphatase; GGT: gamma-glutamyl transferase; AST: aspartate aminotransferase; ALT: alanine aminotransferase

Variable	Result	Reference range	Variable	Result	Reference range
Erythrocytes	4.09 M/µL	4.05 - 5.25 M/µL	Potassium	4.1 mEq/L	3.5 - 5.1 mEq/L
Hemoglobin	11.4 g/dL	12.5 - 15.7 g/dL	Calcium	8.2 mg/dL	8.4 - 10.2 mg/dL
Hematocrit	35%	36.7 - 46.3%	Phosphorus	5 mg/dL	2.3 - 4.7 mg/dL
MCV	85.7 fL	80 - 99 fL	Magnesium	1.8 mg/dL	1.6 - 2.6 mg/dL
MCH	27.8 pg	27 - 33 pg	pH	7.44	7.31 - 7.41
MCHC	32.5 g/dL	32.2 - 36 g/dL	pCO2	33.6 mmHg	41 - 51 mmHg
RDW	17.4%	< 15%	HCO₃⁻	23.3 mmol/L	23 - 29 mmol/L
WBC	16.1 K/µL	3.8 - 11 K/µL	Lactic Acid	1.51 mmol/L	0.36 - 1.39 mmol/L
Eosinophils	0.16 K/µL	0.02 - 0.5 K/µL	Chloride	103 mEq/L	98 - 107 mEq/L
Band Cells	0.32 K/µL	0 - 0.88 K/µL	INR	1.28	0.8 - 1.28
Neutrophils	11.6 K/µL	1.5 - 7.7 K/µL	Albumin	2.6 g/dL	3.5 - 5 g/dL
Lymphocytes	3.06 K/µL	0.8 - 4.9 K/µL	DB	0.12 mg/dL	< 0.5 mg/dL
Monocytes	1.3 K/µL	0.08 - 1.1 K/µL	IB	0.14 mg/dL	< 0.7 mg/dL
Platelets	415 K/µL	140 - 400 K/µL	BT	0.26 mg/dL	0.3 - 1.2 mg/dL
Creatinine	1.51 mg/dL	0.57 - 1.11 mg/dL	ALP	65 U/L	40 - 150 U/L
BUN	40 mg/dL	14.9 - 40 mg/dL	GGT	32 U/L	< 38 U/L
hs-CRP	3.91 mg/dL	⩽ 0.5 mg/dL	AST	32 U/L	5 - 34 U/L
Sodium	137 mEq/L	136 - 145 mEq/L	ALT	42 U/L	< 55 U/L

**Figure 1:**
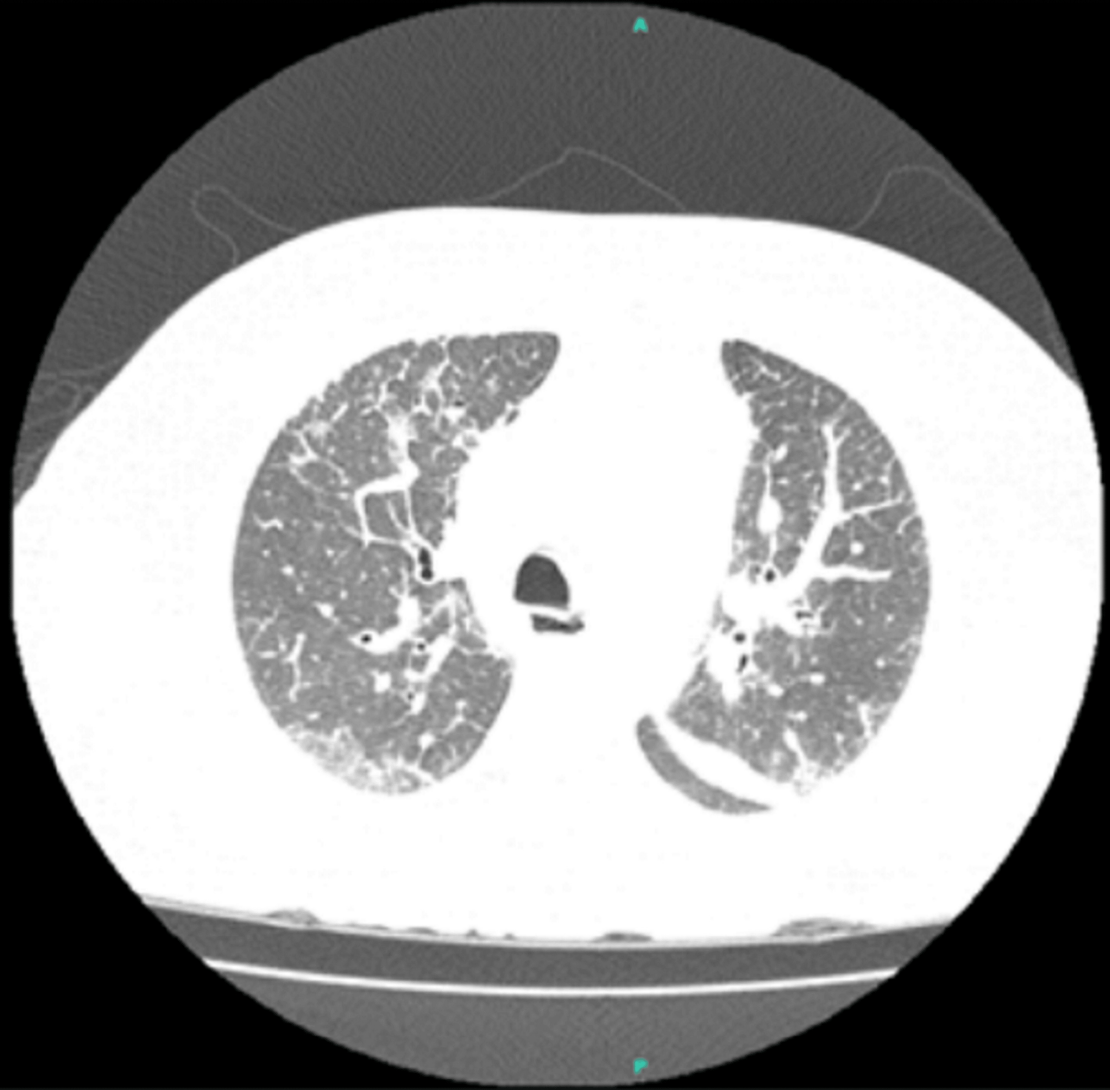
Computed tomography of the lung. The findings were consistent with interstitial lung disease (ILD), including areas of ground-glass opacities and reticular markings, as well as evidence of pulmonary edema characterized by septal thickening and alveolar opacities.

The main diagnostic hypothesis initially for the clinical presentation was a worsening of ILD associated with SSc. The CT scan performed upon admission showed no progression of ILD findings but with signs suggestive of pulmonary edema (Figure 1). The presence of leukocytosis and elevated hs-CRP raised the possibility of a pulmonary infection.

Given the history of prior corticosteroid use and the clinical presentation, the patient was considered at high risk for normotensive SRC, necessitating close monitoring of blood pressure and renal function. The initial management involved discontinuing mycophenolate mofetil due to a suspected infection and initiating empirical antibiotic therapy with levofloxacin 750 mg/day. 

On the third day of hospitalization, September 20, the patient developed severe resting dyspnea, pulmonary congestion, cyanosis in the extremities, and a hypertensive peak of 180/110 mmHg, progressing to cardiogenic shock. Concurrently, renal function worsened, with creatinine levels increasing from 1.51 to 2.29 mg/dL (serial renal function data in Table 2).

**Table 2:** Trend in renal function during hospitalization until peritoneal dialysis. * days on angiotensin-converting enzyme (ACE) inhibitors. Creatine reference range: 0.57 - 1.11 mg/dL; blood urea nitrogen (BUN) reference range: 14.9 - 40 mg/dL

Day	17/09	18/09	19/09	20/09*	21/09*	22/09*	23/09	24/09	25/09	26/09
Creatinine (mg/dL)	1.51	1.78	1.99	2.29	3.25	3.97	4.27	4.26	4.49	4.73
BUN (mg/dL)	40	38	35	39	59		117	133	141	152
Day	29/09*	30/09*	02/10	03/10	04/10	06/10	07/10	08/10	09/10*	10/10*
Creatinine (mg/dL)	4.86	4.88	5.18	5.21	5.43	5.99	6.09	6.15	6.26	6.53
BUN (mg/dL)	142	140	139	130	125	117	109	102	103	

Given the suspicion of decompensated heart failure due to myopericarditis associated with SSc and a potential SRC, treatment was initiated with sodium nitroprusside (0.5 mcg/kg/min) and IV furosemide (80 mg every 12 hours) for congestion management and stringent blood pressure control. Non-invasive mechanical ventilation was also employed to provide respiratory relief.

**Figure 2:**
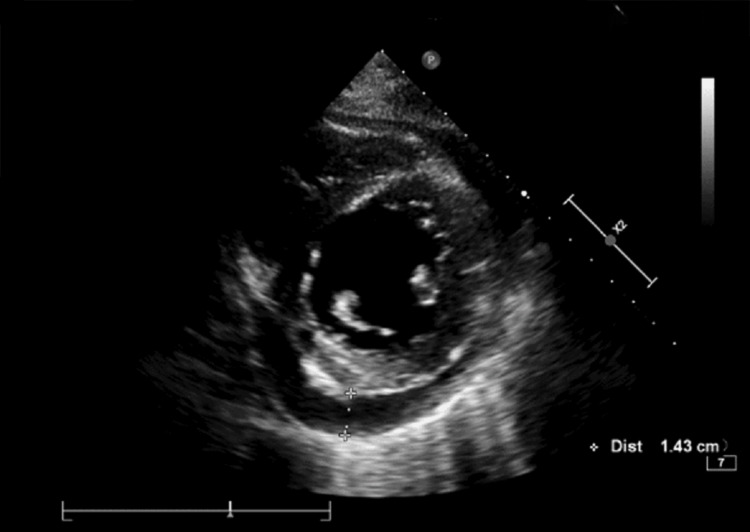
Pericardial effusion visualized on transthoracic echocardiography. Findings of moderate pericardial effusion measuring up to 14 mm overlying the right heart chambers, with no evidence of tamponade.

In the intensive care unit, the ECG revealed sinus tachycardia, while the TTE showed moderate pericardial effusion (up to 14 mm in the right chambers, Figure 2) without signs of tamponade. LVEF was reduced to 23%, with generalized hypokinesia, although internal dimensions were preserved. The right ventricle showed systolic dysfunction, indicated by a tricuspid annular plane systolic excursion (TAPSE) of 13 mm. High-sensitivity troponin I (cTnI) was elevated at 5,059 ng/mL (Table 3). After 24 hours, hemodynamic stabilization was achieved, and captopril 25 mg/day was started.

**Table 3:** Laboratory tests during cardiogenic shock episode. cTnl: troponin I; BUN: blood urea nitrogen; WBC: white blood cells; LDH: lactate dehydrogenase; pCO2: pressure of carbon dioxide; HCO_3_: bicarbonate; CK: creatine kinase

Variable	Result	Reference range	Variable	Result	Reference range
cTnI	5059 pg/mL	< 15.6 pg/mL	Sodium	134 mEq/L	136 - 145 mEq/L
Creatinine	2.29 mg/dL	0.57 - 1.11 mg/dL	Potassium	4 mEq/L	3.5 - 5.1 mEq/L
BUN	39 mg/dL	14.9 - 40 mg/dL	pH	7.47	7.31 - 7.41
Hemoglobin	10.8 g/dL	12.5 - 15.7 g/dL	pCO2	31.4 mmHg	41 - 51 mmHg
Reticulocyte	3.47 %	0.5 - 2.5 %	HCO₃⁻	22.4 mmol/L	23 - 29 mmol/L
WBC	16.2 K/µL	3.8 - 11 K/µL	Lactic Acid	1.8 mmol/L	0.36 - 1.39 mmol/L
Band Cells	0.0 K/µL	0 - 0.88 K/µL	CK	443 U/L	29 - 168 U/L
LDH	580 U/L	125 - 220 U/L			

Although the cardiogenic shock was resolved, renal function continued to deteriorate, with creatinine levels rising to 3.25 mg/dL on September 21. This decline was initially attributed to cardiorenal syndrome in the context of decompensated heart failure, likely secondary to myopericardial involvement related to SSc. Empiric antibiotics were discontinued, and immunosuppressive therapy was resumed with cyclophosphamide (0.5 g/m²), adjusted for a reduced glomerular filtration rate (GFR < 40 mL/min/1.73 m²). The dose was calculated at 850 mg for 12 months, with monthly cycles planned.

Despite the progression of renal dysfunction, angiotensin-converting enzyme (ACE) inhibitors were continued due to a strong suspicion of SRC. However, after three days with ACE inhibitor, creatinine levels rose to 3.97 mg/dL, and mean arterial pressure dropped to 59 mmHg. ACE inhibitor was discontinued due to possible hypoperfusion contributing to renal deterioration, and volume replacement was initiated, guided by hemodynamic parameters.

After being discharged from the intensive care unit, the patient was admitted to the ward on September 29 with progressively worsening renal dysfunction, with creatinine levels rising to 4.86 mg/dL. The patient remained off the ACE inhibitor. Considering the renal protective benefits of ACE inhibitors in patients with suspected SRC, and in light of clinical improvement with normalized blood pressure, captopril was reintroduced at 12.5 mg every 12 hours. However, after three days, on October 2, creatinine levels rose to 5.18 mg/dL, leading to the discontinuation of the ACE inhibitor again.

The investigation of renal dysfunction was further deepened, with emphasis on the possibility of a pre-renal component due to hypoperfusion. The fractional excretion of sodium (FENa) was 5.1%, suggesting an intrinsic etiology, with probable acute tubular necrosis (ATN) secondary to renal hypoperfusion and renal involvement associated with SSc. Urinary tract and renal arteries US showed no alterations that would explain the renal dysfunction. Renal biopsy revealed arterioles with fibrin deposits and concentric "onion-skin" hypertrophy, in addition to ischemic glomerular lesions consistent with hypoperfusion, confirming the diagnosis of SRC.

After the histological diagnosis, captopril was reintroduced on October 9, at a dose of 25 mg/day. Given the patient's difficult vascular access, peritoneal dialysis was chosen as the strategy for renal replacement therapy. The patient started peritoneal dialysis on October 11, performing it daily until her discharge from the hospital. On October 16, due to good tolerance, the dose of captopril was increased to 50 mg/day. On October 24, the dose was adjusted to 75 mg/day, with a favorable clinical response.

The patient was discharged on October 27, with captopril 75 mg/day and peritoneal dialysis. She was instructed to attend outpatient follow-up visits and cyclophosphamide infusion. 

The patient was readmitted on November 19, presenting with mental confusion, tachypnea, and generalized weakness. On examination, vesicular lesions consistent with herpes zoster were observed on the back and in the left inframammary region. In addition, nodular lesions were observed in the left gluteal region, including a deep and painful lesion approximately 6 cm in diameter. Given the sepsis, despite management with antibiotic therapy and hemodynamic support with vasoactive drugs, the patient evolved on November 21 to cardiorespiratory arrest and passed away.

## Discussion

SRC remains a clinical condition associated with high mortality rates despite the introduction and use of ACE inhibitors during the complication period [4,10]. Early deaths, occurring on average within three months of onset, are concerning, with a reported mortality rate of 19% [12]. Additionally, 44% of patients treated with ACE inhibitors during an SRC episode experienced survival of less than six months or required permanent dialysis [13].

The mortality rate of SRC approaches 20% within the first year and exceeds 50% after 10 years of follow-up [4,10]. Additionally, around 63% of patients require dialysis during hospitalization, and 33% of survivors remain on chronic dialysis after five years [4].

An improvement in renal function is typically observed within three years following an SRC episode. Renal involvement is primarily acute, and few patients who do not require dialysis, or who have discontinued it, progress to chronic renal dysfunction [4].

Normotensive SRC accounts for approximately 10% of cases [3], often associated with pericarditis [10], failure to recover renal function necessitating chronic dialysis [4,9,10], corticosteroid use, and thrombotic microangiopathy [11]. Mortality is significantly higher compared to hypertensive SRC, likely due to the more challenging and delayed diagnosis [9-11].

The mortality rate in patients with normotensive SRC remains consistently high in the initial months following the event (42%, compared to 26.2% in the hypertensive group). However, this trend decreases over time, with a higher number of delayed deaths observed in those who initially had hypertension [10]. The mean survival duration in normotensive SRC is shorter than that of the hypertensive cohort (12.7 vs. 99.2 months) [10].

An initial blood pressure reading of <140/90 mmHg at patient admission can independently predict reduced chronic dialysis-free survival [9]. Patients who require chronic dialysis and fail to recover renal function after SRC often exhibit the lowest blood pressure [4].

The increased fatality and poor renal outcomes observed in normotensive SRC can be attributed to delayed diagnosis and challenges in administering optimal treatment doses [3]. Another contributing factor is the higher prevalence of cardiac involvement in these cases [4,14]. Additionally, the absence of hypertension may indicate a decreased pathophysiological reliance on the renin-angiotensin system, which could explain the potential lower effectiveness of ACE inhibitors in normotensive SRC.

Cardiac involvement is an independent risk factor for SRC, with new heart failure increasing the risk approximately sevenfold and pericarditis increasing it fourfold [10]. Pericardial involvement was observed in 33-72% of SSc patients at autopsy and in 15-43% by echocardiography. Symptomatic pericardial effusion was found in only 5-16% of these patients [15]. Among those with symptomatic pericardial effusion, 47% had right heart failure, most also presented with pulmonary hypertension (74%), and a minority with tamponade (17%) [15]. In more than half of the patients, pericardial effusion preceded SRC by an average of six months [16]. Thus, symptoms indicative of pericardial involvement (e.g., dyspnea and chest pain) should alert the attending physician to the potential onset of SRC, particularly in patients with other known risk factors for the condition [7].

Management of pericardial involvement in SSc should be tailored to the underlying cause. Pericardial effusion in these patients primarily results from two factors: the uremic form associated with kidney involvement and the form related to right ventricular dysfunction and pulmonary hypertension [15].

The uremic form of pericardial effusion is more common in younger patients with elevated serum creatinine, and pericardiocentesis is often recommended in these cases. In contrast, pericardial effusion associated with pulmonary hypertension typically affects older individuals, where pericardiocentesis is contraindicated due to the risk of right ventricular decompensation. In these cases, anti-inflammatory therapy is the preferred treatment [15].

Most importantly, the administration of ACE inhibitors should persist in patients with SRC, despite the decline in renal function [9,10], but cautious use of low doses is recommended for normotensive individuals, given the key role of this class in the pathophysiology of SRC [3].

## Conclusions

This report describes a rare case of normotensive SRC with myopericardial involvement. Symptoms suggestive of pericardial involvement in SSc patients should be regarded as predictors of impending SRC. Treatment of pericardial effusion should address the underlying cause. The use of ACE inhibitors in this group is challenging due to the risk of renal hypoperfusion, but they should be prescribed with careful monitoring of renal function, with dose adjustments based on the patient’s response.

## References

[1] van den Hoogen F, Khanna D, Fransen J (2013). 2013 classification criteria for systemic sclerosis: an American College of Rheumatology/European League against Rheumatism collaborative initiative. Arthritis Rheum.

[2] Ingegnoli F, Ughi N, Mihai C (2018). Update on the epidemiology, risk factors, and disease outcomes of systemic sclerosis. Best Pract Res Clin Rheumatol.

[3] Bose N, Chiesa-Vottero A, Chatterjee S (2015). Scleroderma renal crisis. Semin Arthritis Rheum.

[4] Penn H, Howie AJ, Kingdon EJ (2007). Scleroderma renal crisis: patient characteristics and long-term outcomes. QJM.

[5] Steen VD, Syzd A, Johnson JP, Greenberg A, Medsger TA (2005). Kidney disease other than renal crisis in patients with diffuse scleroderma. The Journal of Rheumatology.

[6] Denton CP, Lapadula G, Mouthon L, Müller-Ladner U (2009). Renal complications and scleroderma renal crisis. Rheumatology (Oxford).

[7] Steen VD, Medsger TA, Osial TA, Ziegler GL, Shapiro AP, Rodman GP (1984). Factors predicting development of renal involvement in progressive systemic sclerosis. The American Journal of Medicine.

[8] DeMarco PJ, Weisman MH, Seibold JR (2002). Predictors and outcomes of scleroderma renal crisis: the high-dose versus low-dose D-penicillamine in early diffuse systemic sclerosis trial. Arthritis Rheum.

[9] Teixeira L, Mouthon L, Mahr A (2008). Mortality and risk factors of scleroderma renal crisis: a French retrospective study of 50 patients. Ann Rheum Dis.

[10] Guillevin L, Bérezné A, Seror R (2012). Scleroderma renal crisis: a retrospective multicentre study on 91 patients and 427 controls. Rheumatology (Oxford).

[11] Helfrich DJ, Banner B, Steen VD, Medsger TA Jr (1989). Normotensive renal failure in systemic sclerosis. Arthritis Rheum.

[12] Steen VD, Medsger TA Jr (2000). Long-term outcomes of scleroderma renal crisis. Ann Intern Med.

[13] Steen VD, Costantino JP, Shapiro AP, Medsger TA Jr (1990). Outcome of renal crisis in systemic sclerosis: relation to availability of angiotensin converting enzyme (ACE) inhibitors. Ann Intern Med.

[14] Pei LT, Debajyoti MR, Alvin KH, Archana RV, Alwin HL, Chang YC (2018). Normotensive scleroderma renal crisis. Annals Academy of Medicine Singapore.

[15] Hosoya H, Derk CT (2018). Clinically symptomatic pericardial effusions in hospitalized systemic sclerosis patients: demographics and management. Biomed Res Int.

[16] McWhorter JE, LeRoy EC (1974). Pericardial disease in scleroderma (systemic sclerosis). The American Journal of Medicine.

